# Aboveground Carbon Storage and Its Links to Stand Structure, Tree Diversity and Floristic Composition in South-Eastern Tanzania

**DOI:** 10.1007/s10021-017-0180-6

**Published:** 2017-09-06

**Authors:** Iain M. McNicol, Casey M. Ryan, Kyle G. Dexter, Stephen M. J. Ball, Mathew Williams

**Affiliations:** 1 School of Geosciences, University of Edinburgh, Crew Building, Alexander Crum Brown Road, Edinburgh, EH9 3FF Scotland, UK; 2 Mpingo Conservation and Development Initiative, Kilwa Masoko, United Republic of Tanzania; 3 Present Address: Farm Africa, Dar Es Salaam, United Republic of Tanzania; 4 The National Centre for Earth Observation, Natural Environment Research Council, Swindon, UK

**Keywords:** aboveground carbon storage, tree diversity, Africa, miombo, large trees, biomass–biodiversity relationship, tree species composition, permanent plot, monitoring

## Abstract

African savannas and dry forests represent a large, but poorly quantified store of biomass carbon and biodiversity. Improving this information is hindered by a lack of recent forest inventories, which are necessary for calibrating earth observation data and for evaluating the relationship between carbon stocks and tree diversity in the context of forest conservation (for example, REDD+). Here, we present new inventory data from south-eastern Tanzania, comprising more than 15,000 trees at 25 locations located across a gradient of aboveground woody carbon (AGC) stocks. We find that larger trees disproportionately contribute to AGC, with the largest 3.7% of individuals containing half the carbon. Tree species diversity and carbon stocks were positively related, implying a potential functional relationship between the two, and a ‘win–win’ scenario for conservation; however, lower biomass areas also contain diverse species assemblages meaning that carbon-oriented conservation may miss important areas of biodiversity. Despite these variations, we find that total tree abundance and biomass is skewed towards a few locally dominant species, with eight and nine species (5.7% of the total) accounting for over half the total measured trees and carbon, respectively. This finding implies that carbon production in these areas is channelled through a small number of relatively abundant species. Our results provide key insights into the structure and functioning of these heterogeneous ecosystems and indicate the need for novel strategies for future measurement and monitoring of carbon stocks and biodiversity, including the use for larger plots to capture spatial variations in large tree density and AGC stocks, and to allow the calibration of earth observation data.

**Electronic supplementary material:**

The online version of this article (doi:10.1007/s10021-017-0180-6) contains supplementary material, which is available to authorized users.

## Introduction

Seasonally dry tropical forests and woodlands are the dominant vegetation cover in southern Africa, extending over 4 million km^2^ across 10 countries (Mayaux and others 2004). Across their range, variations in climate, soils and disturbance maintain a structurally and floristically diverse mosaic of habitats, covering a spectrum from open savanna with a dominant grass layer and scattered trees, through open canopy savanna woodland with an understory of grasses and shrubs, to denser woodlands and dry forest (White 1983). The most extensive of these formations are the miombo woodlands, distinguishable from surrounding vegetation types by the dominance of the genera *Brachystegia* and *Julbernardia* (Fabaceae, *Caesalpinioideae*) (Chidumayo 1997). The region as a whole is highly biodiverse and a priority for conservation (Mittermeier and others 2003; Brooks and others 2006), with the miombo woodlands alone thought to harbour an estimated 8500 species of higher plants, including more than 300 tree species (Frost 1996), many of which are endemic to the region. The range of species supported by the ecosystem helps to underpin the livelihoods of an estimated 150 million rural and urban dwellers who rely heavily on the timber, food, medicine and construction materials that the woodlands and forests provide (Ryan and others 2016).

Yet despite their scale and importance for local livelihoods, the ecology and functioning of these seasonally dry ecosystems remain poorly studied in comparison with the more carbon dense moist tropical forests in South America (Fauset and others 2015; Poorter and others 2015), and to a lesser extent, those in Central Africa (Lewis and others 2013). As a result, the miombo eco-region still represents a potentially large, but poorly quantified store of biomass carbon, biodiversity and species endemism (Platts and others 2010; Halperin and others 2016; Ryan and others 2016; Shirima and others 2011; Jew and others 2016). Forest inventory plots with which to quantify these variables are few in number and spatially uneven, typically favouring higher biomass stands and protected areas (Chidumayo 2013; Ribeiro and others 2008; Marshall and others 2012; Willcock and others 2014; Ryan and others 2011; Chidumayo 2002). Thus, many important ecological questions remain poorly resolved, for example, around the magnitude and distribution of aboveground woody carbon stocks (AGC) across these heterogeneous landscapes, and how this relates to patterns in vegetation structure, tree species diversity and composition.

Increasing human pressure linked to resource extraction is currently driving widespread, but uncertain losses of AGC, as well the localised extinction of important tree species (Ahrends and others 2010; Ryan and others 2012; Jew and others 2016). It is therefore important to quantify and reduce uncertainty in our estimates of AGC storage, to better understand future losses, and to underpin carbon sequestration initiatives aimed at mitigating this loss. Plot-level estimates of AGC storage are fundamental for calibrating and interpreting earth observation data, which can then be used to map regional patterns in AGC (Avitabile and others 2016) and its changes over time (Ryan and others 2012).

Measuring and managing ecosystems based on their carbon stocks, particularly under the umbrella of Reducing Emissions from Deforestation and Degradation (REDD+), may also benefit biodiversity research and conservation (Scharlemann and others 2010; Hinsley and others 2014; Ahrends and others 2011). It is therefore useful to quantify how tree diversity and floristic composition co-vary with AGC storage (Hinsley and others 2014) to highlight any important trade-offs and thus inform mutually beneficial conservation schemes (Miles and Kapos 2008; Díaz and others 2009; Venter and others 2009). Such information may also be useful in elucidating a potential functional relationship between AGC storage and tree diversity, which could have additional benefits for conservation if higher tree species diversity also results in higher AGC storage. The majority of the current evidence base for or against a biomass–biodiversity relationship comes from the moist tropical forest biome (Sullivan and others 2016; Chisholm and others 2013), and it is still unclear whether these patterns (or lack thereof) hold true in drier, mixed tree-grass systems.

Despite the comparatively high diversity of the tropical forest biome, recent studies have found that a small number of relatively large trees and species contribute disproportionately to tree abundance and AGC stocks in a variety of moist tropical forest ecosystems (ter Steege and others 2013; Fauset and others 2015; Marshall and others 2012; Bastin and others 2015). The evidence base for similar patterns in the miombo eco-region is limited by a paucity of detailed forest inventories across a range of representative vegetation types and ecosystems (Marshall and others 2012; Frost 1996; Shirima and others 2011). From a measurement perspective, knowing which tree size classes contain most of the carbon and species diversity may also help improve knowledge of how best to design effective data collection protocols which can be used to expand the current plot network (Marshall and others 2012; Réjou-Méchain and others 2014; Bastin and others 2015).

In this paper, we aim improve the knowledge of ecosystem structure and function across these heterogeneous landscapes using data collected from a new network of 25 forest inventory plots in south-eastern Tanzania, which spans a gradient of woody biomass and different vegetation types. Specifically, we explore (1) how patterns in AGC stocks are related to differences in tree size and number, (2) to tree species diversity within plots (*α*-diversity) and (3) to tree species composition.

## Methods

### Study Area and Sampling Strategy

**Figure 1:**
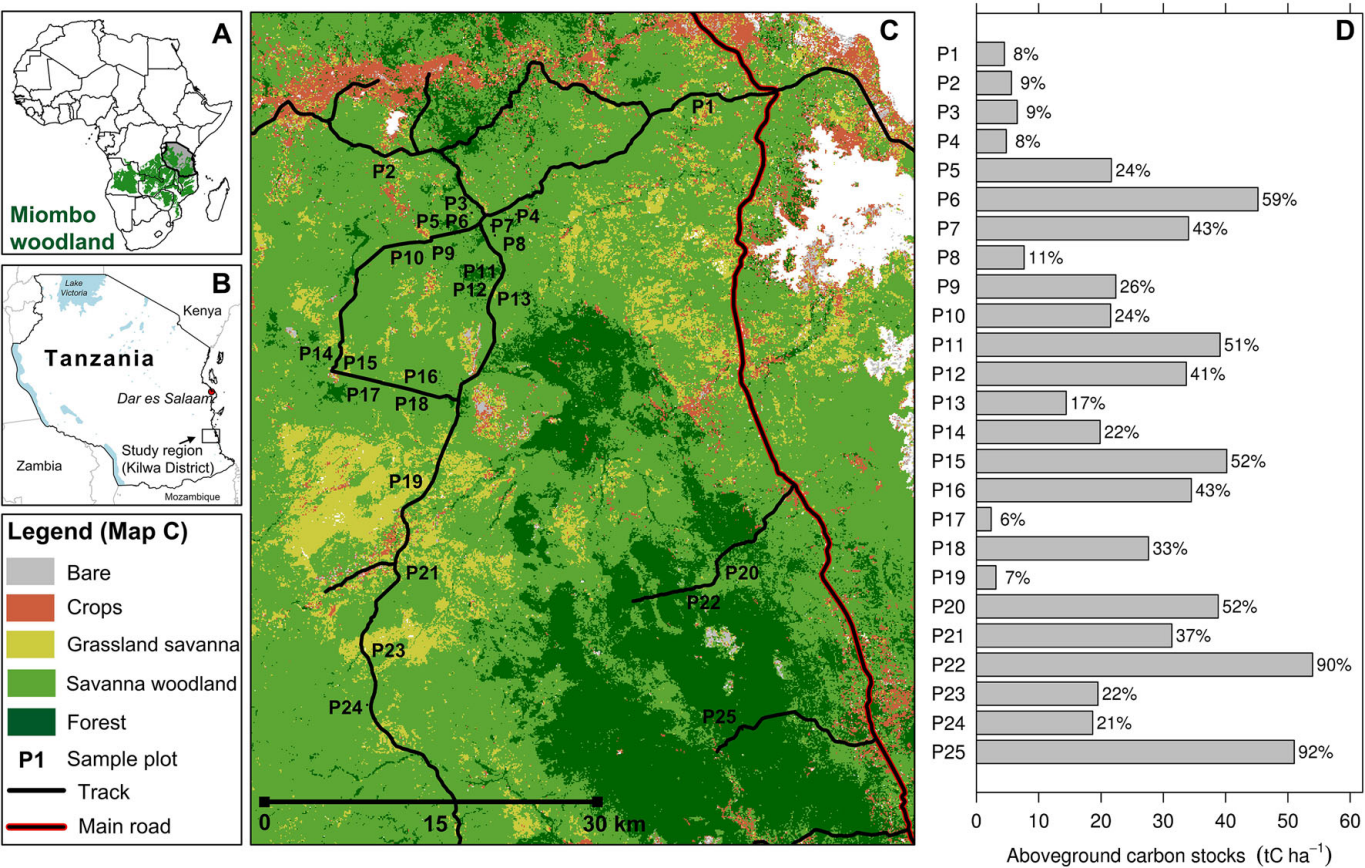
Location of our field plots and associated aboveground woody carbon stock (AGC) and canopy cover estimates. Sub-panel **A** shows the location of Tanzania, and the extent of the miombo woodlands—the dominant vegetation type in our study region, with sub-panel **B** showing the location of our study region. **C** Location of our field plots, and the initial land cover classification used for plot location. **D** The distribution of plot (1 ha) AGC stocks and canopy cover estimates.

The study area is located in Kilwa District in the Lindi Region of south-eastern Tanzania (Figure 1). The estimated mean annual precipitation is 821 ± 350 mm (±SD), with a gradient between the east (wetter) and west (drier) (Tropical Rainfall Measurement Mission, 3B43 product; Huffman and others 2007). Altitude varies from sea level along the coastal plains to the east up to 740 m m.a.s.l along the steep escarpment running north to south dissecting the centre of the district. Approximately 85% of the local population is rural and dependent on natural resources for their livelihoods (Khatun and others 2016). From October 2010–October 2011, permanent sample plots were established at 25 locations, originally stratified by three major vegetation types delineated via a supervised land cover classification, based on Landsat 5 data and 300 in situ visual assessments of land cover, to ensure that potential variations in AGC stocks had been suitably captured (Figure 1). The vegetation types for the original stratification included grass dominated ‘savannas’ with sparse tree cover, savanna woodland (tree-grass mix) and dense woodland and forest (closed tree canopy with no grass cover), with the number of plots measured proportional to the areal extent of each vegetation type. Tree canopy cover was estimated by outlining the crowns of individual trees identified using aerial photographs collected over the plots in October 2010 (Figure 1). Pragmatism played a role in site location, with plots located randomly along the road and track network (Figure 1); however, a 1-km buffer from tracks was enforced to reduce the likelihood of intense human disturbance. For sampling, we utilised a 1-ha (100 × 100 m) sized permanent sample plot in which all trees with a diameter of at least 5 cm were recorded, tagged and spatially located. These 1-ha plots, upon which most of the analyses in this study are based, were nested centrally within a larger 9-ha (300 × 300 m) plot in which only trees larger than 40 cm were recorded. Tree diameter was measured at 1.3 m height above the ground, and if the tree forked below 1.3 m, each stem was measured and counted as one individual. We recorded the local name of each measured tree, and where possible, identified each by their scientific name using collected voucher specimens and published reference guides (Coates-Palgrave and Moll 2002). Where this was not possible, species were identified using a range of local and national species lists (NAFORMA 2011).

### Data Analysis

Aboveground carbon stocks (AGC) were calculated using an allometric model developed in the same administrative region (Lindi model: Mugasha and others 2013), with biomass assumed to be 47% carbon. To address our first question about how variations in AGC stocks are related to differences in stand structure, specifically size and number, trees were binned into 5-cm size classes and the proportional contribution of each size class to the total measured AGC in each plot was calculated. Kolmogorov–Smirnov tests were used to test whether the distribution of plot-level AGC in each size class was statistically different between plots of broadly similar AGC and structure (tree density and canopy cover), under the null hypothesis that the distributions are similar and that variations in AGC storage reflect differences in tree density.

To assess species composition and diversity, we used the species names or genus where known. Where this was not possible, the local name was used instead. In some cases, the use of local names may result in tree species diversity being overestimated if multiple names are used for a single species; however, the more likely scenario is that diversity will be underestimated as the same local name is often used for several species (for example, based on local usage), with some species also likely to be indistinguishable without fertile material leading to some species being conflated (Ahrends and others 2011). To minimise errors due to the former, we used the same botanists for all plots to ensure species identification was consistent across plots. Controlling for the latter is more difficult. However, on average, trees identified only by local name contributed no more than five of the species measured in each plot and thus we consider the likelihood that our diversity measures are subject to meaningful bias to be small. A small numbers of individuals that were not identified to any taxonomic level (0.07% of total inventory) were excluded from the analysis.

Tree species diversity was calculated using three measures: species richness, Fisher’s alpha and rarefied richness. For rarefied richness, we used Mao-Tao individual-based rarefaction analysis. When comparing tree diversity and AGC, diversity is regarded as the independent variable under the assumption that tree diversity has a deterministic effect on AGC at the plot level (due to niche complementarity and selection effects), as opposed to if the axis were reversed, which would assume environmental/disturbance controls on diversity, which we believe are more likely to occur at larger scales than our field plots (Chisholm and others 2013; Woollen and others 2012). Multiple models were fitted to each data set using a variety of functional forms based on ecological theory, including a linear relationship $(y = ax + b$), saturation $(y = ax/\left( {b + t} \right)$, quadratic $\left( {y = ax^{2} + bx + c} \right)$ and a parabolic ricker curve $\left( {y = axe^{ - bx} } \right)$. Model selection was based on minimising the Akaike information criterion (AICc), corrected for small sample sizes, and the residual sum of squared differences.

Diversity measures were taken for all trees (>5 cm) in each 1-ha plot, then again for small trees (5–15 cm), medium sized trees (15–40 cm) and large canopy dominants (>40 cm) separately, with the aim of understanding where most of the tree diversity occurs in these systems. For the analysis of large tree diversity (>40 cm), data from the 9-ha plots were included to allow a suitable number of trees for analysis. Differences in species composition between plots (β-diversity) were calculated using the Bray–Curtis Index of Species Dissimilarity. Overall compositional patterns were visualised using non-metric multidimensional scaling, which was performed using the ‘metaMDS’ function. Permutational multivariate analysis of variance (PerMANOVA) was used to test whether there were significant differences in tree species composition between groups of plots (Anderson 2001). The analysis was repeated separately for small, medium and large trees to test whether composition differed among size classes. Prior to analysis, the raw species abundance data were square root transformed and site standardised to account for the number of trees sampled at each site and to reduce the influence of the most common species (Barlow and others 2007). We used ANOVA and Tukey’s HSD tests to look for significant differences in tree structure and diversity between groups of plots after testing the data for normality using Shapiro–Wilk tests.

To examine how our results (that is, tree diversity and AGC estimates) would have differed had we sampled progressively smaller plots instead of the 1-ha plots, we simulated single sub-plots of varying size (0.1, 0.25 and 0.5 ha) at random locations within each of the 25 × 1 ha plots, with the sub-sampling analysis repeated 1000 times to ensure the full range of possible subsets was achieved. For each subplot, we calculated the tree species richness and AGC density (tC ha^−1^) and compared these as a percentage of the corresponding estimates from the 1-ha plot. For each iteration, we totalled the number of species across the network to show how sampling smaller plots across the entire network would have impacted our estimates of landscape diversity.

All data analyses were performed using the R statistical software version 3.0.2 (R Core Team 2014, http://cran.r-project.org) and the ‘vegan’ package (version 2.0-10; Oksanen 2013).

## Results

### Patterns in Aboveground Woody Carbon Stocks and Stand Structure

In total, we surveyed 13,098 trees (>5 cm) across the 25 one-ha plots, including 10,694 small trees (5–15 cm), 2139 medium sized trees (15–40 cm) and 265 large trees (>40 cm). The surrounding 9-ha plots contained an additional 2069 large trees, highlighting the importance of larger plots for adequate statistical analyses of large trees. AGC stocks in the 1-ha plots ranged from 2 tC ha^−1^ in an area of open grassland savanna to 54 tC ha^−1^ in an area of dense forest (Figure 1), with an overall landscape average of 24 ± 16 tC ha^−1^ (±indicates standard deviation throughout).

This gradient in AGC stocks is associated with clear changes in both tree density (72–1511 trees ha^−1^; Spearman’s rho, *R* = 0.95, *P* < 0.001) and tree canopy cover, with areas of <10% cover—broadly consistent with the FAO definition of ‘other wooded lands’ (FAO 2001)—storing <10 tC ha^−1^ (*n* = 7), with plots in more open canopy savanna ‘woodlands’ (10–45%) storing 15–35 tC ha^−1^ (*n* = 12), and plots in more closed canopy ‘forests’ (>50%) containing >40 tC ha^−1^ (*n* = 6). Large trees contributed around one-third (32 ± 18%) of plot AGC, despite comprising only 2.6 ± 2.2% of the trees in each plot. Overall, half of the total measured biomass (across the 1-ha plots) was stored in the 484 largest trees, which comprised 3.7% of the total trees measured.

**Figure 2:**
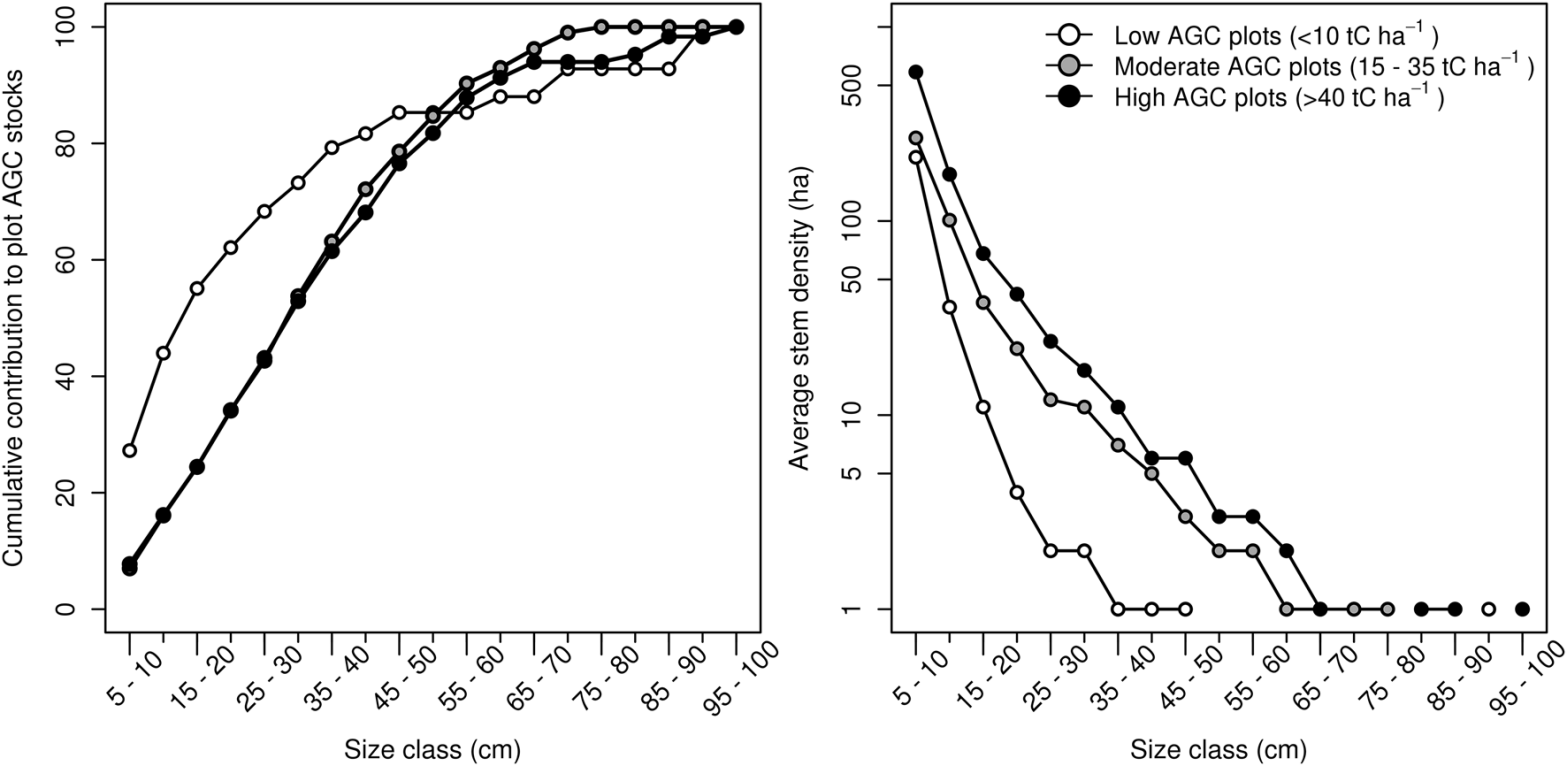
**A** Cumulative percentage of AGC stocks contributed by different tree size classes within plots of similar AGC and canopy cover; **B** the average number of trees within each size class. *Each data point* represents the average contribution of plots within each group.

The distribution of carbon stocks among tree size classes differed significantly between our low AGC density plots (<10 tC ha^−1^) and those with a moderate and high AGC density (Kolmogorov–Smirnov; *P* = <0.001 in both cases). In the low AGC density, typically grassland savanna plots, the majority of AGC (42%) was contributed by the smallest diameter classes (5–15 cm) (Figure 2), whereas in moderate density savanna ‘woodlands’ and higher AGC density ‘forest’ plots, the proportion of AGC stored in small trees was relatively low (~15%), despite the greater number of trees in these areas. There were no significant differences in the distribution of AGC among different size classes between our moderate and high AGC density plots (*P* = 0.51), despite a clear trend towards greater tree size (that is, >80 cm DBH) at the upper end of the gradient, where these very large trees had a disproportionate contribution to plot AGC (~10%) relative to their abundance (1 ± 1 ha^−1^) (Figure 2).

### Patterns in Tree Species Composition and Diversity

We identified 158 morphospecies across the 25 × 1 ha plots by their local species name, of which 91 were fully identified to species level (57%) and a further 16 to genus (10%), with 32 taxonomic families present. In the surrounding 9-ha plots (>40 cm DBH trees only), 79 morphospecies were identified, including 26 not found in the 1-ha plots, with 54 (68%) of these identified to species level, and 3 (4%) to genus, with a further three families represented. In both 1- and 9-ha plots, the identified taxa contributed 96% of the total measured trees and AGC across all sites. The data presented in the following sections are from the 1-ha plots unless otherwise stated.

**Figure 3:**
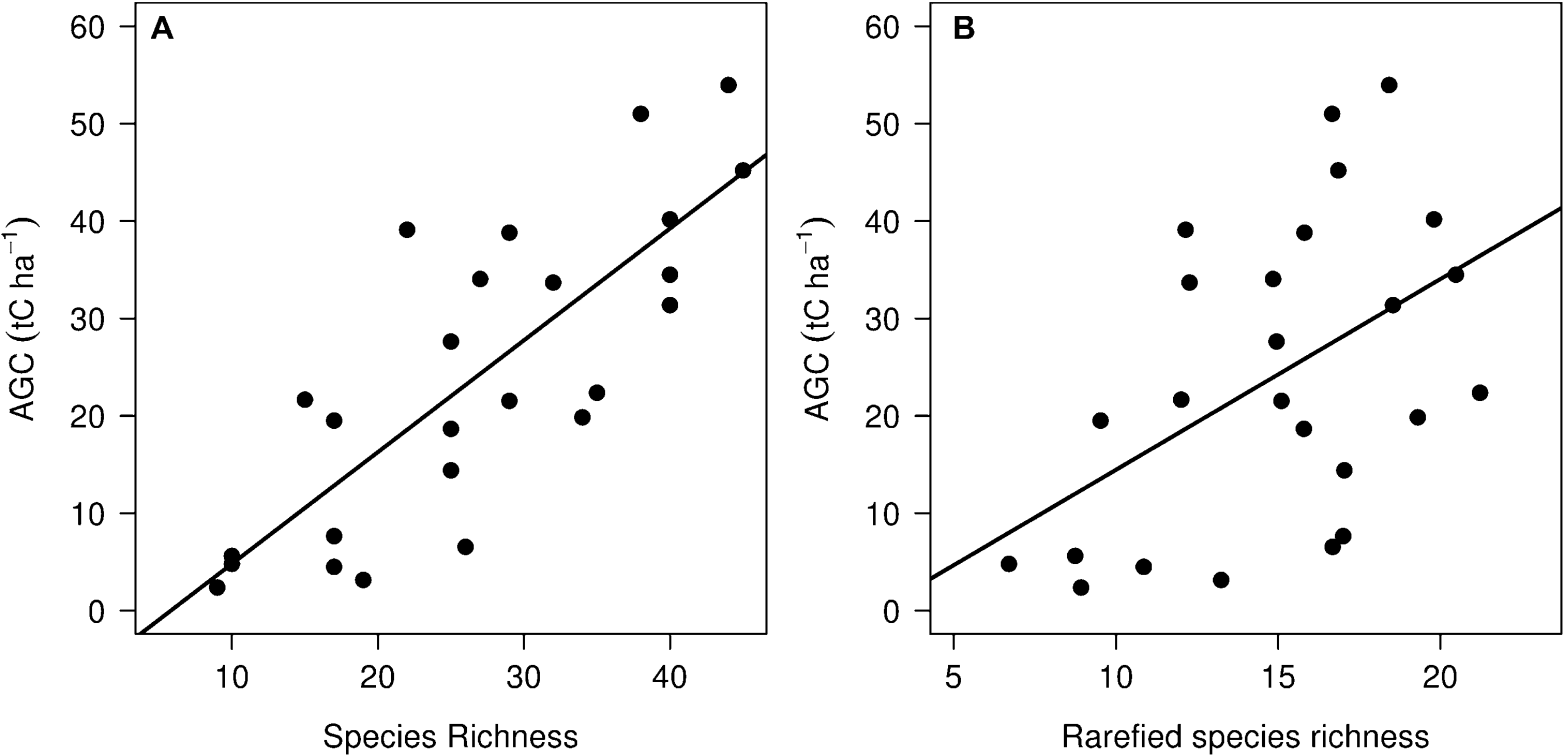
Relationships between tree species richness and aboveground woody carbon stocks. Ordinary least squares (OLS) regression models are fitted to the data; **A** tree species richness (*y* = 1.15–6.67, *r* ^2^ = 0.63, *P* = <0.001) and **B** rarefied richness (*y* = 1.95–5.12, *r* ^2^ = 0.22, *P* = 0.01).

Tree species richness ranged from 9 to 45 per plot with both richness and Fisher’s α significantly higher in the moderate and high AGC density plots compared to the lowest density plots (ANOVA + Tukey HSD, *P* < 0.01) (Table 1). The results were the same when comparing small, medium and large trees separately (Table 2). Tree species richness exhibited a positive linear relationship with AGC storage (*r* ^2^ = 0.63, *P* < 0.001) (Figure 3). The significant trend was maintained when controlling for tree density (rarefied richness), though the relationship was markedly weaker (*r* ^2^ = 0.22, *P* = 0.01) (Figure 3), indicating that differences in tree density partly drive this relationship.

**Table 1:** Top 5 Dominant Species Within Plots of Broadly Similar AGC Stocks Ranked by Their Contribution to the Total Carbon Stock and Total Tree Abundance

Rank	Low AGC (0–10 tC ha^−1)^	Low to moderate AGC (15–25 tC ha^−1^)	Moderate to high AGC (25–40 tC ha^−1^)	High AGC (>45 tC ha^−1^)
*AGC (tC* *ha* ^−*1*^ *)*				
1	*Diospyros quiloensis*	*Dalbergia melanoxylon*	*Julbernardia globiflora*	*Hymenocardia ulmoides*
2	*Sclerocarya birrea*	*Pseudolachnostylis maprouneifolia*	*Brachystegia spiciformis*	*Hymenaea verrucosa*
3	*Combretum apiculatum*	*Julbernardia globiflora*	*Combretum apiculatum*	*Rytigynia* sp.
4	*Dalbergia melanoxylon*	*Combretum apiculatum*	*Burkea africana*	*Pteleopsis myrtifolia*
5	*Burkea africana*	*Brachystegia spiciformis*	*Diplorhynchus condylocarpon*	*Euphorbia nyikae*
% of total	53.6	44.3	60.9	49.7
*Stocking density (trees ha* ^−*1*^ *)*				
1	*Combretum apiculatum*	*Diplorhynchus condylocarpon*	*Diplorhynchus condylocarpon*	*Hymenocardia ulmoides*
2	*Spirostachys africana*	*Combretum apiculatum*	*Combretum apiculatum*	*Suregada zanzibariensis*
3	*Acacia nilotica*	*Dalbergia melanoxylon*	*Pseudolachnostylis maprouneifolia*	*Euphorbia nyikae*
4	*Burkea africana*	*Pseudolachnostylis maprouneifolia*	*Hymenocardia ulmoides*	*Uvaria lucida*
5	*Bauhinia petersiana*	*Bridelia scleroneura*	*Julbernardia globiflora*	*Strychos spinosa*
% of total	62.8	55.4	64.7	52.0
*n* plots	7	7	8	3
Species richness	15 (6)	26 (8)	32 (7)	42 (4)
Fisher’s α	4.2 (2.3)	6.4 (2.3)	7.5 (1.7)	8.2 (0.7)
Total species richness	56	74	95	87
Bray–Curtis	0.77 (0.11)^a^	0.69 (0.14)^b^	0.52 (0.10)^b^	0.61 (0.10)^c^
Number of unique species	9	10	26	32

**Figure 4:**
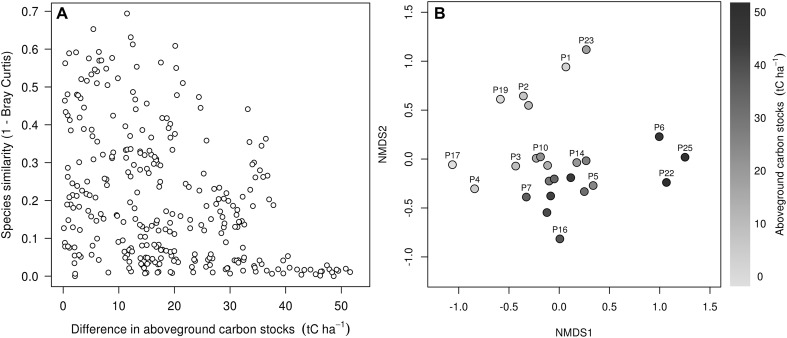
**A** Plot-pair differences in tree species composition with differences in plot-level AGC stocks; **B** NMDS ordination based on the Bray–Curtis Index which is used to uncover the main compositional patterns across the gradient in AGC storage.

Plots with a moderate and high AGC density are further separated to better highlight changes in tree species dominance over the gradient, particularly in our three highest AGC plots (> 60% tree canopy cover) which are marked out as floristically distinct from other high AGC plots (Figure 4). Additional information includes the mean tree species richness and Fisher’s α in each plot (±SD), the total number of species recorded in each group, as well as the number that are unique to each group, and the average species dissimilarity between plots [Bray–Curtis Index (±SD)]. The letters in superscript next to the Bray–Curtis index indicate the results of the PerMANOVA which tested whether trees species composition significantly differed between groups of plots.

**Table 2:** Diversity Indices for Group of Plots Separated by Broad Size Class

Size class	Small trees (5–15 cm DBH)	Medium trees (15–40 cm DBH)	Large trees^a^ (40 cm + DBH)
*Low AGC*			
Species richness	14 (7)	6 (3)	7 (4)*
Fisher’s *α*	3.2 (2.0)	3.8 (3.7)	3.4 (2.0)
Bray–Curtis Index	0.77 (0.11)^a^	0.89 (0.12)^a^	0.77 (0.12)^a^
*Moderate AG* ***C***			
Species richness	22 (6)	15 (5)	15 (5)*
Fisher’s *α*	5.5 (1.7)	6.0 (2.3)	4.9 (1.6)
Bray–Curtis Index	0.66 (0.14)^b^	0.67 (0.13)^b^	0.64 (0.17)^b^
***High AGC***			
Species richness	28 (9)	19 (4)	17 (4)*
Fisher’s *α*	6.2 (1.1)	5.6 (1.7)	5.0 (1.1)
Bray–Curtis Index	0.73 (0.14)^c^	0.74 (0.15)^b^	0.74 (0.16)^b^

As in Table 1 information includes the average species richness and Fisher’s *α* (±SD) for different size classes within each plot. The Bray–Curtis Index is used to highlight difference in floristic composition within plots. The letters in superscript indicate the results of the PerMANOVA which tested whether the composition of small, medium and large trees significantly varied between groups of plots.

^a^Includes the measured trees from the 9-ha plot meaning that comparisons of large tree species richness are only valid between groups, and not between size classes due the larger sample area for large trees compared to medium and smaller trees.

Euphorbiaceae was the dominant family across the plot network, comprising 39% of the total measured AGC and 17% of trees, followed equally by Combretaceae and Fabaceae (each ~21% of AGC and ~11% of trees), and Apocynaceae (12; 17%). Familial dominance differed among vegetation types with trees in the family Euphorbiaceae more common in areas with an AGC density greater than 40 tC ha^−1^ (39; 24%), with those in Fabaceae proportionally more dominant in lower biomass grassland savannas and savanna woodlands (39; 21%), compared to the ‘forests’ (25; 6%) where they were few in number, but large. This pattern was also true for potentially nodulating legumes (Caroline Lehmann and others unpubl. data.) which were almost absent in high AGC areas, yet gradually more common as AGC stocks decreased, comprising 40% of trees in low density plots.

A small number of species were both abundant and widespread, with 8 species collectively contributing over 50% of the trees measured, including *Diplorhynchus condylocarpon* (15.9% of all trees; *n* plots = 17), *Combretum apiculatum* (10.6%; *n* = 21), and to a lesser extent, *Hymenocardia ulmoides* (9.9%; *n* = 8) and *Pseudolachnostylis maprouneifolia* (3.6%; *n* = 16*)*. A similar level of dominance was observed when assessing species contributions to the total carbon stock, with just 9 species, including the four aforementioned species, containing over half (52.5%) of the total AGC. The remaining biomass dominant species were *Julbernardia globiflora* (15.4% of total measured biomass), *Brachystegia spiciformis* (7%), *Burkea Africana* (4.5%), *Pteleopsis mytifolia* and the priority conservation and timber species*, Dalbergia melanoxylon*, with the remainder either commonly used for charcoal (*P. myrtifolia*), or occasionally harvested for timber. A similar level of species dominance was observed within each of the broad vegetation types, with approximately 5 species contributing over half of the AGC stocks and trees (Table 1).

The large majority of species were considerably less abundant, with 49 species (31% of total) contributing fewer than 50 individuals. Many of the recorded species were restricted to particular habitats, with nine restricted to the low AGC plots, 36 to plots with a moderate AGC density, with 32 species only found in the three highest AGC ‘forest’ plots (Figure 4). Species turnover (β-diversity) among plots was therefore relatively high, with some areas of similar AGC found to contain entirely different species assemblages (Figure 4). The lowest AGC plots were the most heterogeneous (Table 1), as shown by the NMDS ordination plot (stress = 0.12, *n* dimensions = 3) and were floristically distinct to both the moderate and high AGC plots, both when considering all tree together (>5 cm) (PerMANOVA, *P* < 0.001; Figure 4; Table 1) and small, medium and large trees separately (Table 2).

Despite the wider range of AGC storage, we observed a greater compositional similarity among the moderate density ‘woodland’ plots (15–40 tC ha^−1^), which tend to be dominated terms of AGC contribution by two of the defining miombo woodland species—*J. globiflora* and *B. spiciformis*—and in number by *D. condylocarpon* and *C. apiculatum* (Table 1). At the upper end of the gradient, species characteristic of wet miombo woodland and coastal forest was common, including *Suregada zanzibariensis* and *Hymenaea verrucosa*. This shift in tree composition is reflected in the NMDS plot with the three highest AGC plots—two of which were located at relatively high elevations along an escarpment (Figure 1)—exhibiting clear differences in composition (Figure 4), both when considering all trees together, and when comparing trees in different size classes (PerMANOVA; *P* < 0.001; Tables 1, 2).

## Discussion

### Links Between Vegetation Structure and Aboveground Carbon Storage

Our landscape-level estimates of aboveground carbon (AGC) stocks (24 ± 16 tC ha^−1^) are similar to those recorded using similar approaches in Mozambique by Ryan et al. (2011) (21 ± 11 tC ha^−1^) and Woollen and others (2012) (21 ± 10 tC ha^−1^), but lower than the regional average (28.7 ± 19.1 tC ha^−1^) (Ryan and others 2016) which includes many plots from protected areas which are unlikely to be representative of the wider miombo eco-region. Our lowest AGC plots, defined as areas with a tree canopy cover (%) and AGC stock (tC ha^−1^) of less than 10, were characterised by a lower tree density, with the majority of trees (80%), and thus AGC (42%) contained in smallest size classes (5–15 cm DBH), as is common with more disturbed systems. The results highlight the obvious importance of maintaining a low DBH threshold (that is, 5 cm) in lower biomass stands in order to capture and quantify the majority of AGC stocks.

In the more carbon dense savanna woodlands and dry forest plots, a greater proportion of AGC was contained in larger trees, with the relative proportion contained in different size classes statistically similar between plots in moderate (10–35 tC ha^−1^) and high AGC (>40 tC ha^−1^) stands. We therefore conclude that the variations in AGC stocks between these areas are due to differences in tree abundance in each size class, although there is some evidence to suggest that these differences may also reflect the greater density of very large trees (≥80 cm) in forests, which typically numbered only one per hectare in the most carbon dense ‘forest’ plots (>50% canopy cover), yet contributed on average 8% of the measured AGC. These very large trees were comparatively rare in the low density, typically grassland savanna plots; however, where a very large tree was present on a plot (>94.9 cm, *Diospyros quiloensis*), its contribution to the total measured AGC was considerable (50%).

The concentration of biomass in a small number of trees has been previously observed in other moist forest ecosystems (Bastin and others 2015; Fauset and others 2015; Slik and others 2013) and has clear implications for the development of rapid, low-cost forest monitoring protocols. In more wooded areas (that is, >10 tC ha^−1^/% canopy cover), large trees—that is, those larger than 40 cm—comprised approximately 40% of the biomass measured in each plot, with half the plot AGC contained in the top 4.9% of trees (range 2.7–9%; *n* trees = 9–64; minimum DBH = 24–46 cm). These results are consistent with the results of Bastin and others (2015) who detected a similar concentration (that is, 50%) of plot biomass in a similar proportion of trees (~5% of total) across Central African moist forests. Similar results were also found across an identical plot network in the miombo woodlands of Mozambique (Ryan 2009; Ryan and others 2011), where approximately 50% of plot AGC was contained in trees larger than 40 cm DBH, suggesting this is a common feature of miombo-dominated woodlands. Our results contrast with those of Marshall and others (2012) who found that in the moist forests of the Eastern Arc Mountains, trees larger than 40 cm stored a much higher proportion (75–80%) of plot AGC.

The tendency towards greater tree size in plots at the upper end of the gradient may be due to their location at moderate to high elevations (Marshall and others 2012), suggesting a possible topographic, and/or edaphic influence on AGC storage (Woollen and others 2012). These plots were also more remote from human populations (Figure 1), meaning that historically lower levels of disturbance (human and ‘natural’) in these areas may have allowed larger trees to persist and AGC to accrue over longer periods. In the moderate AGC density plots (10–35 tC ha^−1^), we found no trees larger than 75 cm DBH, yet in the surrounding 9-ha plots, several trees (*n* = 12) surpassed this limit (max. 112 cm), suggesting that in some cases, even 1-ha plots are unable to fully capture the stem size distribution of woodlands (Anderson and others 2009). This in turn may lead to high sampling errors when scaling AGC estimates across the landscape (Fisher and others 2008; Réjou-Méchain and others 2014), or remote sensing data of coarser resolutions than the plots, such as the European Space Agency’s Biomass mission, which will operate at a resolution of 4 ha (Scipal and others 2010). This mismatch again highlights the importance of sampling on a sufficiently large scale, either through sampling many smaller plots, or a few larger plots, to account for the inherent patchiness of these ecosystems and presence of rare large trees.

### Relationship Between AGC Storage, Tree Species Diversity and Composition

The inclusion of biodiversity as a co-benefit in carbon sequestration projects necessitates an assessment on how the two co-vary to assess potential trade-offs, or co-benefits of conservation initiatives. From an ecological perspective, examining these linkages along with the extent to which certain species contribute to carbon storage in these systems, will help with efforts to reveal a more deterministic relationship between these two variables, and likely resilience of these ecosystems to future changes in land use (Hinsley and others 2014).

We find clear differences in tree species composition along our AGC gradient, with the lowest AGC stands and our three highest biomass plots marked out as being floristically distinct from the spatially extensive, and moderate AGC density miombo-dominated ‘woodlands’. The compositional patterns suggest that the associated variations in AGC storage along the gradient may be partially explained by differing functional traits between the dominant species in each area, such as their maximum tree height (Nzunda and others 2014) and shade tolerance. In contrast, the noted compositional similarities among the moderate density plots mean it is unlikely that differences in composition are driving the within-vegetation type heterogeneity in AGC storage. Our results therefore suggest that compositional/functional differences may be more important in explaining the variation between, rather than within vegetation types.

Despite this diversity in tree species composition, we find that total tree abundance and biomass is skewed strongly towards a relatively few locally dominant species (Shirima and others 2011), with 8 species (5.7% of the total) accounting for over half the measured trees and 9 species for greater than 50% of biomass. A larger degree of biomass- and stem-‘hyperdominance’ is found in the more diverse rainforests of both Amazonia (Fauset and others 2015; ter Steege and others 2013), and to a lesser extent, Central Africa (Bastin and others 2015), although these results are derived from much larger regional plot networks. In our study area, the relatively large proportion of biomass located in such a small number of trees (90% is contained in 38 species) suggests that most biomass productivity in these seasonally dry ecosystems is also channelled through a relatively small number of tree species. The additional finding that greater than 50% of the biomass is contained in moderate to high value timber suitable trees also highlights the future sensitivity of woody carbon stocks, and potentially productivity, in this area to logging and/or charcoal production (Ahrends and others 2010).

From a conservation standpoint, our finding that more carbon dense areas also harbour the greatest tree species diversity suggests a ‘win–win’ scenario for forest conservation projects operating under the umbrella of REDD+. Among the recorded species were a number that are endemic to the remaining fragments coastal forest in the region, including *H. verrucosa* and *Uvaria kirkii*, which is recorded as ‘Near Threatened’ on the IUCN red list. Lower biomass stands, particularly the miombo (*Julbernardia*—*Brachystegia*)-dominated ‘woodlands’, also contained a relatively diverse assemblage of trees, including a number of high value timber species, such as *Pterocarpus angolensis* which is commercially extinct in many parts of Tanzania (Jew and others 2016) and classified as ‘Near Threatened’, and the priority conservation species *Dalbergia melanoxylon*. A large number of species were also found to be constrained to either moderate or high density stands resulting in localised patterns of species endemism. As such, the ‘win–win’ scenario indicated by our results does not mean that comparatively low biomass areas should be excluded from conservation efforts, as these areas may retain many locally and biologically important species, particularly in the understory (that is, woody plats < 5 cm), and herbaceous layers, as well as in faunal communities (Murphy and others 2016), none of which were sampled in this study.

The preservation of biodiversity may have additional benefits if higher tree species diversity also results in higher AGC storage. Our finding of a positive relationship between diversity and AGC storage is consistent with other observational studies from both the miombo eco-region (Shirima and others 2015) and other forests globally (Ruiz-Jaen and Potvin 2010; Ruiz-Benito and others 2014; Vilà and others 2007; Maestre and others 2012; Liang and others 2016; Poorter and others 2015). This positive relationship is consistent with theories of (1) niche complementarity, where a higher tree species richness leads to a more functionally diverse community and thus greater resource capture and biomass production; and (2) selection effects, which posit that in already dense stands there is a greater chance that one or a few highly productive species are present (Fridley 2001). The absence of any clear saturation in the relationship at higher biomass levels, which would be suggestive of species redundancy or competitive exclusion, indicates that relatively dense patches of vegetation are still capable of efficiently utilising available resources to allow many species and high AGC stocks to coexist, suggesting that some form of complementarity or facilitation is operating in these areas. Yet despite the statistical significance of the relationships, there was considerable variability in tree diversity between plots, particularly after accounting for differences in tree density. Recent studies from moist tropical forests indicate that diversity controls on AGC storage operate at much smaller scales than the ones observed here (~0.1 ha) (Chisholm and others 2013; Poorter and others 2015; Sullivan and others 2016), which may explain the lack of explanatory power. An alternative explanation is that the greater diversity of tree species at higher AGC densities is the result of more heterogeneous environmental conditions within these areas, leading to greater species turnover related to habitat specialisation in certain patches. High AGC may also occur in areas that have fewer major disturbances, allowing species less adapted to disturbance to persist.

A full assessment of the biomass–diversity relationship over larger scales will help answer questions over whether tree diversity does indeed have a mechanistic effect on AGC storage and productivity in these systems, which is important for understanding how changes in biodiversity will affect these important ecosystem functions (Liang and others 2016). It is also unclear whether more diverse tree communities help to create greater diversity across multiple trophic levels, and whether these communities also increase the ecosystem services provided to humans such as timber resources and medicinal products (Maestre and others 2012), both of which are important areas of future research.

### Potential Implications for Future Tree Measurement and Monitoring

The need to acquire data on AGC stocks has taken on added significance due to the rise in carbon sequestration initiatives such as REDD+. The collection of species data also needs to be included in any future measurement campaign to allow co-variation between AGC and biodiversity to be explored in the context of forest conservation (Venter and 2009; Liang and others 2016; Ahrends and 2011). Expanding the current network of permanent inventory plots is a necessity, and a standardised methodology based on existing data sets is crucial to rapidly facilitate the establishment of new plots in the region and aid cross-plot comparisons. To date, no studies have presented a clear view on the most appropriate and efficient strategy (that is, sample size, plot size, appropriate DBH threshold) for accurately measuring carbon stocks and/or biodiversity in savanna woodlands (that is, Baraloto and others 2013), a fact which is evidenced by the wide variety of sampling methodologies used to for tree measurement (Ribeiro and others 2008; NAFORMA 2010; Chidumayo 2013; Ryan and others 2011; Willcock and others 2014). The RAINFOR manual has provided some consistency based on data collected in Amazonian forests (Phillips and others 2009; Phillips and others 2003); however, there is no equivalent methodology for the dry tropics which are very different in terms of their tree structure, diversity and composition (Fauset and others 2015; ter Steege and others 2013). The results here provide some insights in how sampling could be tailored in future to suit the aims of a given project and its available resources.

For example, we show that in more wooded areas (>10 tC ha^−1^, >10% canopy cover), where stem size distribution is broadly consistent across sites, measuring only those trees larger than 10 cm DBH would have captured on average 93% of the total AGC in each plot, yet would have required measuring 40% of the trees, or skipping on average approximately 600 trees ha^−1^ in denser woodlands and dry forests (>40 tC ha^−1^) and approximately 275 trees ha^−1^ in more open canopy savanna woodlands (10–35 tC ha^−1^). Raising the threshold to 15 cm would still have captured 86% of the total AGC stocks in only 20% of the trees. We suggest that such an approach would be ideal for conducting rapid inventories of AGC, such as for the calibration of earth observation data.

Measuring for biodiversity and species composition would have very different requirements with 50% of the species sampled here likely to be missed when measuring trees larger than 10 cm. These species are likely to be among the rarest; therefore, sampling at a higher DBH threshold will have little value when assessing the biodiversity or conservation value of these areas. Our results also suggest that for a given site, the use of smaller inventory plots (that is, <0.5 ha) (Willcock and others 2014; NAFORMA 2010; Shirima and others 2015), which are ideally suited for rapid sampling and often used for species measurement across the tropics (Stohlgren and others 1995; Baraloto and others 2013; Phillips and others 2003), are potentially more sensitive to species clustering and/or likely to exclude rare tree species (Baraloto and others 2013). For example, in the 9-ha plots, we find 26 species not in the 1-ha plots, despite measuring only those trees larger than 40 cm in these areas, suggesting that even 1-ha plots fail to fully capture the species diversity at certain sites. We explored this potential issue further by sub-sampling the 1-ha plots which showed that the use of smaller plots would have captured on average 36 ± 13% (0.1 ha), 53 ± 14% (0.25 ha) and 71 ± 14% (0.5 ha) of the plot-level tree species richness. Hence, smaller plots clearly sample a smaller proportion of tree species for a given site than the 1-ha plots (Phillips and others 2003). However, sampling 0.5-ha plots instead of the 1-ha plots at each site would still have captured a large majority (80 ± 2%) of the tree species found across the entire 1-ha network in only half the sample area, highlighting that the use of smaller plots may be more efficient for gathering large-scale floristic data. The issue of many potentially rare tree species being missed in the smaller plots could be avoided if sampling a larger number of these across the wider landscape; however, the physical and financial challenges associated with repeat plot establishment and accessing typically remote areas may outweigh the costs associated with establishing a smaller number of well stratified larger plots (Baraloto and others 2013). Based on our data set, it is unclear which of these sampling strategies (“few large” vs. “many small” plots) is more appropriate for accurately and cost effectively capturing tree species diversity and composition in these areas. Such information will be important for facilitating conservation planning and implementation and will likely require the intensive (sub)-sampling of very large plots to properly address this question (Baraloto and others 2013).

The issue of plot size has additional importance for measuring biomass, with smaller plots more likely to either overestimate, or completely miss the presence of rare, large trees, thus creating significant small scale variations in AGC stocks (Réjou-Méchain and others 2014; Fisher and others 2008; Chave and others 2004). Indeed, we find that even the 0.5-ha plots produce highly variable AGC densities (tC ha^−1^) relative to the corresponding 1 ha values (5–95th percentile; 40–120%), tending towards underestimation (median = 90%) (Chave and others 2003). These sampling errors were exacerbated when using progressively smaller sub-plots, with 0.25 ha (25–150%) and 0.1 ha (14–200%) plots generating an ever-larger range of possible AGC values relative to the 1-ha estimates. The 0.1-ha plots also produced anomalously high values above 100 tC ha^−1^ where a large tree(s) is present. For this reason, we would caution against the use of very small plots (that is, <0.25 ha) for measuring biomass as they can create large uncertainties on AGC stocks for a given site. However, if replicated in sufficient number, smaller plots may still be suitable for estimating the average AGC density across the landscape, although such estimates may be less precise (Chave and others 2004).

This issue of plot size has clear relevance when considering the suitability of the plots for the calibration of remotely sensed data; particularly radar (for example, ALOS PALSAR) and LiDAR sensors, which in future will be the primary method for upscaling ground based AGC estimates to the landscape scale. Smaller plots (for example, <0.25 ha) tend to be unsuitable for this purpose due to the aforementioned scaling issues, but also their larger relative geo-location errors which may be of similar size to the field plot (Ryan and 2012). As a result, AGC stocks measured in larger plots are often found to exhibit a much stronger relationship with the remotely sensed observation (Carreiras and others 2013; Réjou-Méchain and others 2014; McNicol 2014; Robinson and others 2013; Mauya and others 2015). The mismatch in spatial scale between many of the current field inventory plots (Shirima and others 2011; Willcock and others 2014; Ryan and others 2011) and the larger pixels of future sensors such as the European Space Agency’s Biomass mission (4 ha) (Scipal and others 2010) also has the potential to introduce considerable errors when scaling plot even our 1 ha AGC values to the size of the radar pixel (Réjou-Méchain and others 2014). The use of higher DBH thresholds would allow for larger areas (that is, >1 ha) to be sampled in a more time and cost-efficient manner, as was achieved in this study with the 9-ha plots which were typically sampled in two-third of the time taken to sample the 1-ha plots; however, this would clearly be at the detriment of biodiversity assessment. As shown here, the sampling of large plots (that is, >1 ha) also has the additional benefit of capturing of suitable of number larger trees, which will be useful for the analysis of large tree mortality.

The development of a standardised field protocol that appropriately incorporates measurements of tree species diversity and aboveground carbon stocks, but is also suitable for the calibration of earth observation data, is urgently needed in order to ensure the best use of time and resources. For this reason, we would suggest that larger sample plots (that is, ≥1 ha) should be favoured where possible to capture potentially important variations in large tree densities, and thus AGC stocks, whereas at the same time, allowing the plots to be used as a calibration points for earth observation data, and facilitating cross-project comparisons (that is, RAINFOR). These plots may form part of nested sampling strategy to account for the different data requirements, including the use of smaller plots (for example, 0.5 ha) for the sampling of tree species diversity, and potentially even smaller plots for sampling the understory and herbaceous layer, which was not sampled at all in this study, yet is a major store of diversity in these ecosystems (Murphy et al. 2016).

## Electronic supplementary material

Below is the link to the electronic supplementary material.

Supplementary material 1 (CSV 16 kb)
